# Real-World Utilization of Tissue-Free ctDNA MRD Testing in Non-Small Cell Lung Cancer Across Asia-Pacific and the Middle East: A Descriptive Cohort Analysis and Illustrative Case Series

**DOI:** 10.3390/cancers18203296

**Published:** 2026-10-13

**Authors:** Chun-Hui Lee, Suyog Jain, Lun-Che Chen, Nir Peled, Gerardo H. Cornelio, Kenny Kim Pong Ng, Hsiao-Yu Jen, Katherina Oh, Orielle Kyra B. Castro-Maddela, Vince Nigel Blanco, Chun-Ming Tsai

**Affiliations:** 1 Department of Oncology, National Cheng Kung University Hospital, Tainan 704, Taiwan; chunhui811012@gmail.com; 2 Medical Affairs Department, Guardant Health Pte Ltd., Singapore 138567, Singapore; ajen@guardanthealth.com (H.-Y.J.); koh@guardanthealth.com (K.O.); 3 Department of Internal Medicine, National Taiwan University Hospital Hsinchu Branch, Hsinchu 300, Taiwan; b94401048@gmail.com; 4 The Helmsley Cancer Center, Shaare Zedek Medical Center, Jerusalem 9103102, Israel; nirp@szmc.org.il; 5 Cancer Institute of St. Luke’s Medical Center, Bonifacio Global City, Taguig 1634, Metro Manila, Philippines; ghcornelio@gmail.com; 6 Medical Oncology, reVIVE Oncology and Cancer Centre, Hong Kong, China; kimpong1@gmail.com; 7 St Luke’s Medical Center, Quezon City 1112, Metro Manila, Philippines; okcmaddela@stlukes.com.ph (O.K.B.C.-M.); vnmblanco@stlukes.com.ph (V.N.B.); 8 Department of Oncology, Taipei Veterans General Hospital, Taipei City 112201, Taiwan; doc3006a@gmail.com

**Keywords:** circulating tumor DNA, molecular residual disease, non-small cell lung cancer, surveillance, Asia-Pacific

## Abstract

**Introduction:** Circulating tumor DNA (ctDNA)-based molecular residual disease (MRD) testing is emerging as a tool to refine relapse risk and guide surveillance after curative-intent therapy for non-small cell lung cancer (NSCLC). Tissue-free (plasma-only) MRD assays may improve feasibility where tumor tissue is limited. Prospective and trial-embedded studies have established that ctDNA MRD detected after curative-intent therapy is prognostic for recurrence in NSCLC, but how tissue-free MRD testing is deployed in routine care in Asia-Pacific and the Middle East (AMEA) has not been described. We characterized ordering patterns and first-test positivity in an AMEA cohort. In this descriptive cohort (159 NSCLC patients; 257 samples), 40.9% of patients underwent more than one MRD test, with a median interval of 4.9 months between the first two tests; first-test MRD positivity was 15.7% (25/159; 95% CI 10.9–22.2) with rates of 0% (0/9; 95% CI 0.0–29.9), 8.8% (5/57; 95% CI 3.8–18.9), 21.5% (17/79; 95% CI 13.9–31.8) and 21.4% (3/14; 95% CI 7.6–47.6), respectively, across stages I, II, III, and oligometastatic stage IV disease. No hypothesis testing was performed, and the stage I and oligometastatic stage IV subgroups are small, so the confidence intervals overlap widely and apparent between-stage differences should not be interpreted as a stage effect. **Case Presentation:** Six illustrative cases involving patients with stage II–IVB describe the use of MRD testing in routine clinical care. The cases were selected purposively, not consecutively, to span the contexts in which the results were interpreted: a positive result shortly before radiographic nodal recurrence (Case 1); a positive result during long-term adjuvant osimertinib preceding isolated mediastinal relapse (Case 2); a transient positive result with subsequent clearance and radiographic regression (Case 3); a positive result that prompted PET-CT revealing occult metastatic disease (Case 4); serially negative results concordant with sustained radiographic remission (Case 5); and a positive result that prompted comprehensive genomic profiling identifying an actionable KRAS G12C alteration (Case 6). **Conclusions:** Across Asia-Pacific and the Middle East, tissue-free MRD testing in NSCLC was used primarily during surveillance and often serially; first-test positivity was 15.7% (95% CI 10.9–22.2) overall, with wide and mutually overlapping confidence intervals across stages. The cases illustrate that MRD results may prompt diagnostic evaluation when interpreted alongside imaging and other clinical findings, but they are purposively selected, and treatment changes that followed a result are reported as description, not as evidence of benefit. No assay performance characteristic is estimated. These findings are hypothesis-generating; prospective, MRD-directed trials with standardized timepoints, thresholds, confirmatory strategies and pre-specified clinical actions are needed before MRD results can guide treatment.

## Simple Summary

Tissue-free (plasma-only) ctDNA molecular residual disease (MRD) testing is used in some clinical settings after curative-intent therapy for patients with non-small cell lung cancer (NSCLC) in routine clinical practice to support relapse-risk assessment and surveillance. We describe real-world utilization across Asia-Pacific and the Middle East and present six illustrative cases that show how serial MRD results can be considered alongside standard monitoring in clinical decision-making. This is a descriptive utilization study. It reports who was tested, when and how often, and it does not compare outcomes between tested and untested patients or evaluate whether MRD-guided management improves recurrence-free or overall survival.

## 1. Introduction

Despite advances in surgery, radiotherapy, and systemic therapy, recurrence after curative-intent treatment remains a major cause of mortality in NSCLC. Imaging-based surveillance does not directly detect molecular relapse at its earliest phase. ctDNA-based MRD testing offers a noninvasive approach to detecting residual disease and may complement imaging for recurrence monitoring [1,2].

Perioperative treatment strategies are evolving rapidly in NSCLC (e.g., adjuvant osimertinib for epidermal growth factor receptor (EGFR)-mutated disease, adjuvant atezolizumab in selected patients after chemotherapy, and neoadjuvant immune checkpoint inhibitor plus chemotherapy). MRD testing is being explored to refine risk stratification and to support treatment optimization in clinical studies. In resected NSCLC, ctDNA-based MRD has consistently been associated with recurrence risk, whereas treatment-selection utility remains under investigation: LUNGCA-1 found that postoperative MRD positivity was strongly associated with relapse (hazard ratio, HR 11.1) and that MRD-positive patients appeared to benefit from adjuvant therapy [3]; TRACERx detected ctDNA within 120 days of surgery in 25% of patients, including 49% of those who later relapsed, with serial monitoring identifying additional impending recurrences [2]; the longitudinal study by Qiu et al. showed that postsurgical ctDNA positivity was associated with worse recurrence-free survival (RFS) (HR 4.0) and preceded imaging relapse by ~88 days [4].

An exploratory, post hoc MRD analysis from ADAURA found that MRD detection preceded imaging-based disease-free survival (DFS) events by a median of 4.7 months; DFS or MRD event-free outcomes favored osimertinib [5]; IMpower010 ctDNA analysis showed ctDNA was prognostic and cleared in approximately 62% of patients receiving chemotherapy, with atezolizumab improving DFS in PD-L1-positive disease [6], an effect reported in full in the primary trial publication [7]; and a meta-analysis (10 studies, 1859 patients) reported a strong association between MRD positivity and worse progression-free survival (PFS) (HR 11.19) and overall survival (OS) (HR 6.34), supporting ctDNA MRD as a prognostic and early-relapse biomarker, though prospective MRD-guided treatment strategies remain under investigation [8].

Real-world data on MRD testing in patients with lung cancer are limited. One such dataset using a tissue-free MRD test (Guardant Reveal) in early-stage NSCLC patients from Asia and the Middle East (AMEA) was based on 104 NSCLC patients (168 samples) with documented stage and at least one tissue-free MRD test. It reported that forty-five patients (43%) underwent serial testing, with a median interval of 5.3 months between the first and second tests. Overall positivity at any timepoint was 12.5%, and first-test positivity was 11.5%, increasing with stage (0% in stage I, 10.71% in stage II, 14.81% in stage III, and 25% in stage IV). Among all tests ordered for stage II and stage III disease, most were ordered during surveillance (76.06% in stage II, 73.75% in stage III) [9,10].

Read together, these studies establish two things and leave a third open. ctDNA MRD detected after curative-intent therapy is strongly prognostic for recurrence in NSCLC, and MRD detection frequently precedes radiological relapse [2,3,4,5,6,8]. Whether acting on an MRD result changes outcome is unresolved, and is the subject of ongoing prospective work. What is almost entirely undescribed, however, is implementation: which patients clinicians actually test, where in the care pathway the test is ordered, how often it is repeated, and what proportion of tests return a positive result outside the fixed sampling schedules of a clinical trial. Existing MRD evidence is derived predominantly from tumor-informed assays studied in North American, European and Chinese cohorts, whereas tissue-free assays are attractive in settings where archival tissue is limited or exhausted. Implementation questions are particularly relevant in AMEA, where tissue availability, reimbursement pathways, surveillance imaging practice and access to perioperative systemic therapy differ both from North America and Europe and between territories within the region.

This analysis therefore has two deliberately descriptive objectives: to characterize how tissue-free ctDNA MRD testing is ordered in routine NSCLC care across AMEA, including timing relative to surgery and the frequency of serial testing, and to report first-test ctDNA positivity by stage in this cohort. An accompanying case series illustrates the clinical contexts in which results were interpreted. The analysis was not designed to estimate assay sensitivity, specificity or predictive value, to compare outcomes between tested and untested patients, or to determine whether MRD-guided management alters recurrence-free or overall survival, and it should not be read as addressing any of those questions. The earlier report from this program [9,10] described an overlapping subset of 104 patients; the present cohort extends that dataset to 159 patients and 257 samples and adds longitudinal, case-level clinical detail that the earlier abstracts did not contain.

Patients with oligometastatic stage IV disease were retained rather than excluded because, in the routine practice this analysis sets out to describe, they are tested for the same purpose as patients with resected stage I–III disease: local consolidative or definitive therapy is delivered with the intention of rendering the patient disease-free, and MRD testing is then ordered to assess residual disease. Excluding them would have misrepresented how the assay is being used. However, because their biology, treatment intent, and baseline recurrence risk differ materially from stage I-III disease, this subgroup (*n* = 14) is reported separately at every point and is not pooled with stages I–III.

## 2. Materials and Methods

### 2.1. Plasma Sample Analysis Methods

MRD analysis was performed using the analytically validated, commercially available tissue-free Guardant Reveal assay (Guardant Health, Inc., Redwood City, CA, USA) [9]. As previously reported, the Guardant Reveal assessment includes a review of approximately 20,000 differentially methylated regions (DMRs), including those most strongly associated with lung cancer, and classifies each sample as ctDNA detected (positive) or not detected (negative) using a predefined statistical threshold based on the likelihood that the methylation patterns are tumor-derived.

For this real-world utilization analysis, methylation-based MRD status was abstracted from the Guardant Reveal report as a binary result: ctDNA detected (MRD-positive) or ctDNA not detected (MRD-negative). The authors performed no additional methylation calling, thresholding, or laboratory validation for this study; analytical validation and assay performance characteristics are described in the published Guardant Reveal validation studies [11].

### 2.2. Patient Cohort

Patients with lung cancer from the AMEA region who underwent the Guardant Reveal test as part of their routine clinical care were included in this analysis. MRD tests can be ordered for stage I, stage II, stage III, and oligometastatic stage IV post-surgery or post-adjuvant therapy.

All samples were processed by a single central laboratory, so every result reported here was generated on one platform using one reporting algorithm and one result-classification threshold; inter-laboratory variability is therefore not a source of heterogeneity in this dataset. Clinical heterogeneity, by contrast, is substantial and is intrinsic to the question being asked. The number of patients contributed by individual territories is too small to support territory-level comparison, and no such comparison is attempted.

Testing was ordered entirely at the discretion of the treating physician. There was no protocol-specified indication, no mandated sampling schedule, no eligibility screening and no central review of ordering decisions; the assay-specific indications recorded above define only when the test may be ordered, not when it should be. Test requisition forms did not capture the clinical reasoning behind any individual order. We are therefore unable to describe differences in ordering thresholds between physicians, between institutions or between health systems, and any such variation is embedded within, rather than adjusted out of, the utilization patterns reported here. This is a limitation of the data source, but it is also the object of study: these are the ordering patterns that arise when the test is available and unconstrained by protocol.

First, 159 consecutive lung cancer patients with 257 longitudinal samples between July 2023 and July 2026 from the AMEA region, with test results based on the then-current commercially available version of Guardant Reveal, were retrospectively queried. Data were analyzed for patients with a valid test result and a documented clinical stage. Testing in this program is indicated after completion of definitive, curative-intent therapy. For stage I-III disease, this was surgical resection with or without neoadjuvant and/or adjuvant systemic therapy, or definitive chemoradiotherapy in patients who did not undergo resection (as in Case 3); for oligometastatic stage IV disease, it was resection and/or local ablative therapy delivered with definitive intent, in addition to systemic therapy. A date for surgery was recorded on the requisition form for the majority of the patient samples. Completeness of resection (R status), the intent documented in the operative record, and the delivery of definitive non-surgical therapy could not be independently verified from requisition forms. It therefore cannot be confirmed that every patient in the cohort underwent resection with curative intent, and this should be borne in mind wherever post-resection timing is reported. Based on the timing of the MRD test order, we classified them into post-resection (defined as the first test ordered within 12 weeks of surgery) or during surveillance (defined as the first test ordered more than 12 weeks after surgery). The 12-week boundary is an operational convention adopted for consistency of reporting, not a biologically validated threshold, and no biological transition is claimed to occur at that point. It was chosen because it comfortably encompasses the postoperative landmark windows used in the principal NSCLC MRD studies (typically 3–6 weeks after surgery, and within 120 days in TRACERx [2]), because it allows time for initiation of adjuvant chemotherapy before a test is reclassified as surveillance, and because the same boundary was applied in the earlier report from this program [9,10], preserving comparability. The boundary was applied uniformly across stages. We acknowledge that this is a simplification: baseline recurrence risk, the duration of adjuvant or consolidation therapy, and surveillance intensity all differ by stage, so a test at 11 weeks does not carry the same clinical meaning in stage I as in stage III or in oligometastatic stage IV disease. For this reason, all timing results are presented stage by stage rather than pooled, and the absolute median and range of timing are reported alongside the dichotomy so that readers can assess the effect of the cut-off directly. We did not test the sensitivity of the reported proportions to alternative thresholds. Clinical factors (age, sex, stage at diagnosis, date of surgery, adjuvant therapy status, and timing of MRD testing) were extracted from test requisition forms submitted when the test was ordered. Additional information from selected patients was included to describe the case reports, including subsequent clinical management and the context in which test results were considered. For the six cases, written informed consent for publication was obtained by the treating physicians. The six cases were not randomly sampled, are not consecutive, and are not representative of the cohort. They were selected purposively by the investigators to span the range of clinical contexts in which a tissue-free MRD result was interpreted in routine practice: a positive result shortly before radiographic recurrence (Case 1); a positive result arising during long-term adjuvant targeted therapy (Case 2); a transient positive result with subsequent clearance (Case 3); a positive result that prompted imaging revealing occult metastatic disease (Case 4); serially negative results concordant with radiographic remission (Case 5); and a positive result that prompted comprehensive genomic profiling identifying an actionable alteration (Case 6). Eligibility required that the treating physician was an investigator on this analysis, that the clinical record was complete enough to reconstruct the sequence of tests, imaging, and treatment, and that written consent for publication had been obtained. This process necessarily favors cases in which the MRD result proved informative, and therefore over-represents concordance between MRD status and subsequent clinical course. The cases carry no information about how often such sequences occurred in the cohort, and no inference about assay sensitivity, specificity or predictive value should be drawn from them. The study was conducted in accordance with the Declaration of Helsinki and approved by the Advarra Institutional Review Board (protocol code: Pro00034566/CR00218935, approved on 9 September 2019 and was last renewed on 7 July 2023).

### 2.3. Data Extraction and Statistical Analysis

This study is a retrospective, non-interventional real-world utilization analysis of Guardant Reveal MRD testing performed as part of routine clinical care. All analyses were descriptive.

The study cohort consisted of the first 159 consecutive lung cancer patients with a documented cancer stage and a valid MRD test result, comprising 257 total samples collected longitudinally between July 2023 and July 2026. For patient-level analyses of ctDNA positivity, the first MRD test result was used (*n* = 159). For utilization analyses, the timing of test orders was summarized at both the sample level (all tests) and at the patient level (first test). Continuous variables were summarized using medians and ranges, and categorical variables were summarized using counts and percentages. No hypothesis testing was planned or performed; therefore, no *p*-values or thresholds for statistical significance were applied. Each blood sample corresponded to a single clinical test result (no technical replicates). When multiple tests were available for a patient, these represented serial samples obtained at different timepoints rather than replicate measurements. Missing data were handled by complete-case analysis with no imputation. The denominator for every variable is the number of patients or samples for which that variable was recorded, and any denominator that differs from the cohort total is stated alongside the corresponding result. Samples that did not yield a reportable result (assay failure, insufficient plasma quantity, or otherwise non-reportable) were excluded during cohort assembly and are not among the 257 samples analyzed. Because the dataset was assembled from test requisition forms rather than from linked longitudinal clinical records, no systematic follow-up was available for the cohort: there was no protocol-defined follow-up period, no censoring date, and no ascertainment of recurrence, progression or death. Time-to-event analysis was therefore impossible, and no estimate of sensitivity, specificity, positive or negative predictive value can be derived from these data; none was attempted. Recurrence and outcome information was available only for the six patients described in Section 4, and was obtained from the treating physician. Data extraction from test requisition forms and descriptive analyses were performed using Microsoft Excel (Microsoft^®^ Excel^®^ for Microsoft 365 MSO (Version 2609 Build 16.0.20430.20032) 64-bit).

## 3. Results

### 3.1. Demographics

A total of 159 patients (257 samples) with at least one MRD test result and a cancer stage documented by the ordering physician were included in the analysis. Demographic details are shown in Table 1.

### 3.2. Test Utilization and ctDNA Positivity by Cancer Stage

**Figure 1:**
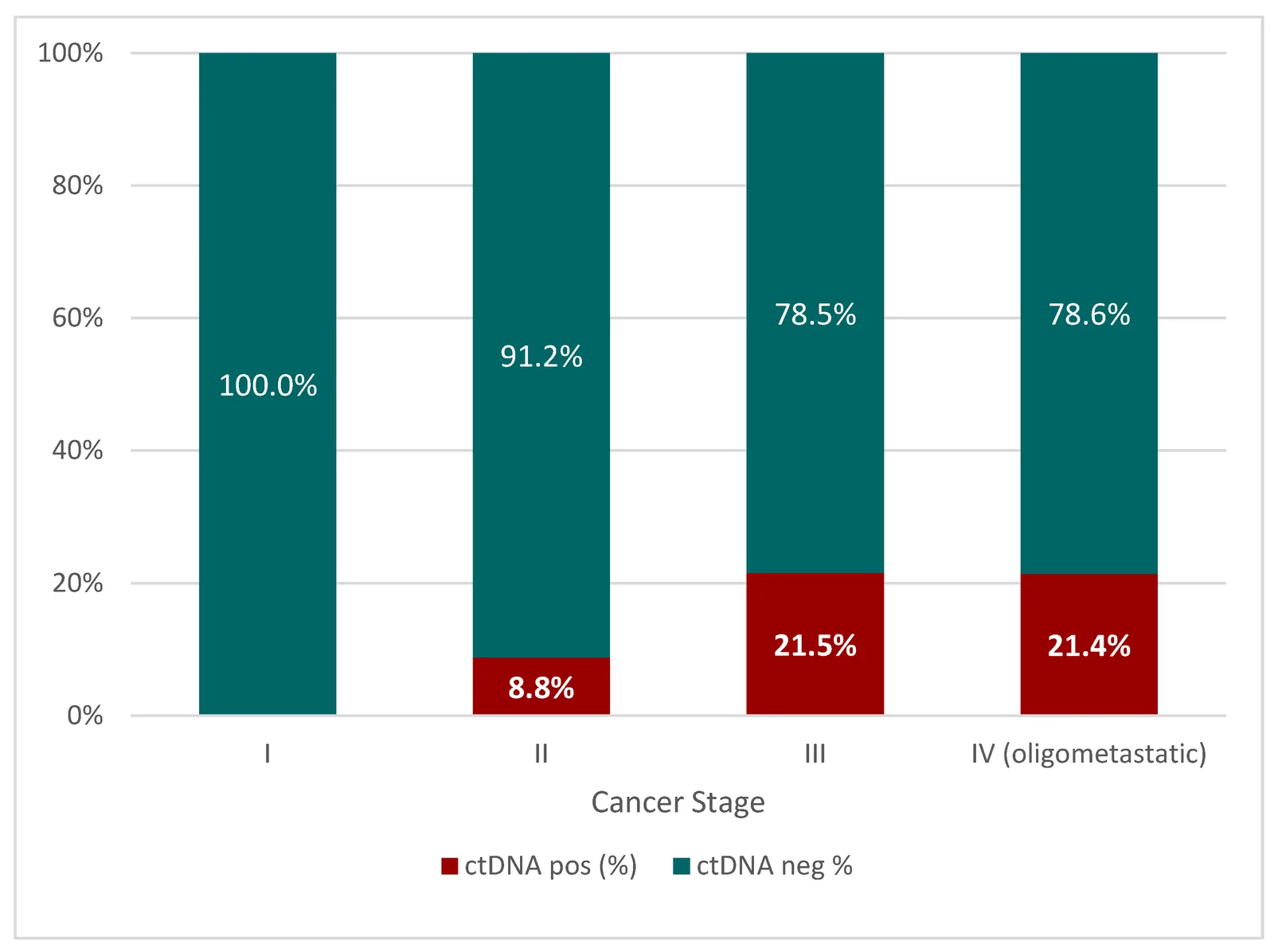
ctDNA positivity at the first MRD test by cancer stage: stage I (*n* = 9; 0 positive); stage II (*n* = 57; 5 positive); stage III (*n* = 79; 17 positive); and oligometastatic stage IV (*n* = 14; 3 positive).

Of 159 patients, 40.9% (65/159; 95% CI 33.5–48.6) underwent serial testing, with more than one MRD test ordered. The median interval between the first and second MRD test was 4.9 months. ctDNA positivity (MRD-positive status) was defined based on the result of the first MRD test. At the test level, 14.8% (38/257; 95% CI 11.0–19.6) of all samples were reported as ctDNA-positive; because patients could contribute repeated samples, this percentage is not a patient-level prevalence estimate. At the first MRD test, ctDNA was detected in 15.7% of patients (25/159; 95% CI 10.9–22.2) across all disease stages. First-test positivity by stage was 0% in stage I (0/9), 8.8% in stage II (5/57; 95% CI 3.8–18.9), 21.5% in stage III (17/79; 95% CI 13.9–31.8) and 21.4% in oligometastatic stage IV (3/14; 95% CI 7.6–47.6) (Figure 1). Pooled across stages I-III, first-test positivity was 15.2% (22/145; 95% CI 10.2–21.9).

Serial testing was common but was not schedule-driven. Among the 65 patients with more than one test, the median interval between the first and second test was 4.9 months. Because the decision to retest rested with the treating physician, intervals varied widely, and a patient with a positive first result may plausibly have been retested sooner than one with a negative result. Any apparent cohort-level trajectory would therefore be confounded by informative sampling. For this reason, we did not analyze negative-to-positive conversion or positive-to-negative clearance rates across the cohort. Serial results are shown at the individual level in Cases 3, 5 and 6, where the sampling context for each draw is known.

### 3.3. Stratification of MRD Test Order Timing by Cancer Stage

**Figure 2:**
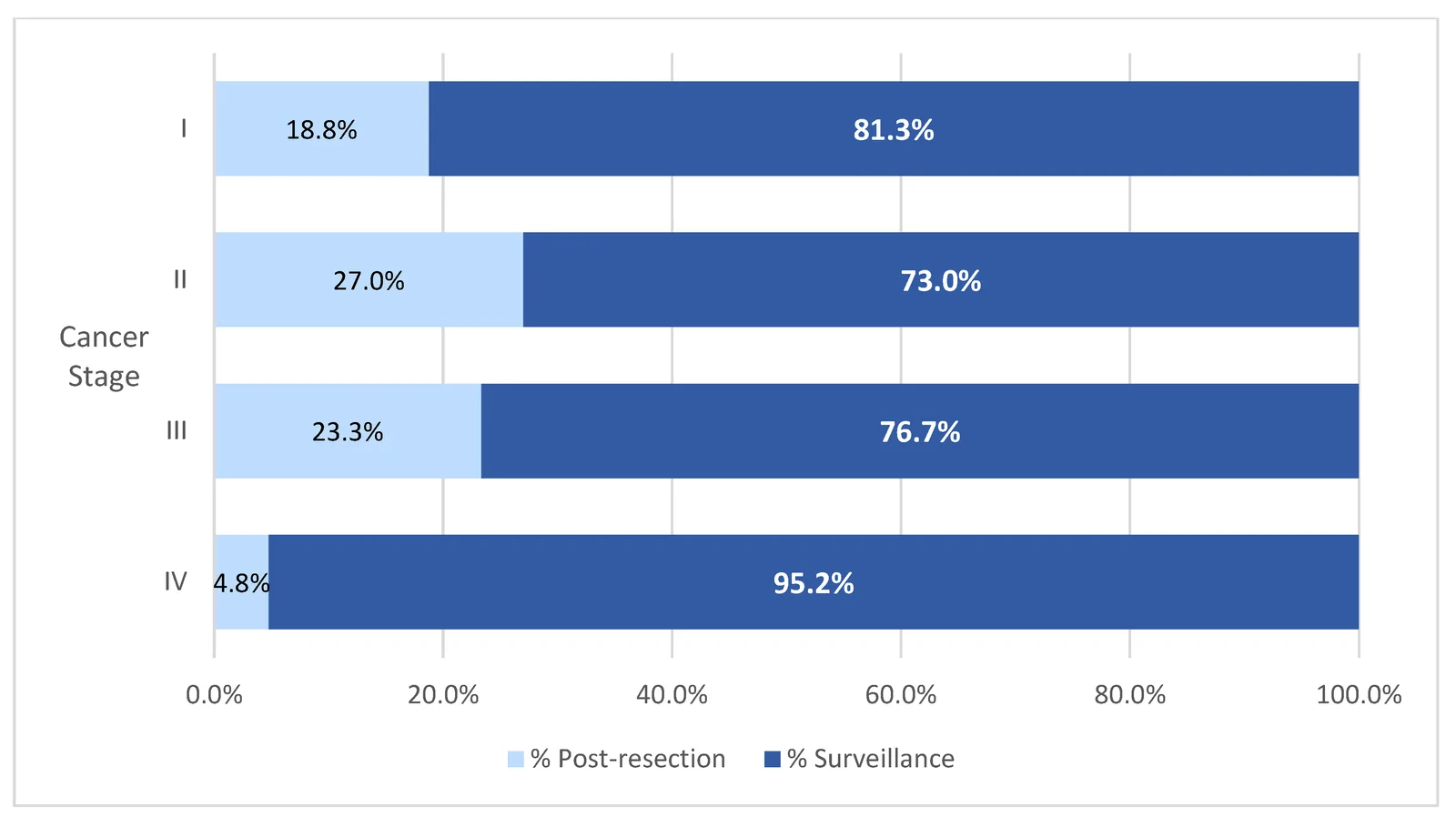
Overall timing of MRD test order for stage I (*n* = 16), stage II (*n* = 100), stage III (*n* = 120), and oligometastatic stage IV (*n* = 21).

Across all 257 samples, the majority of MRD tests were ordered during surveillance, irrespective of tumor stage (as defined in Methods). The proportion of tests ordered in surveillance was 81.3% in stage I, 73.0% in stage II, 76.7% in stage III and 95.2% in oligometastatic stage IV (Figure 2).

### 3.4. Timing of the First MRD Test Order by Cancer Stage

**Figure 3:**
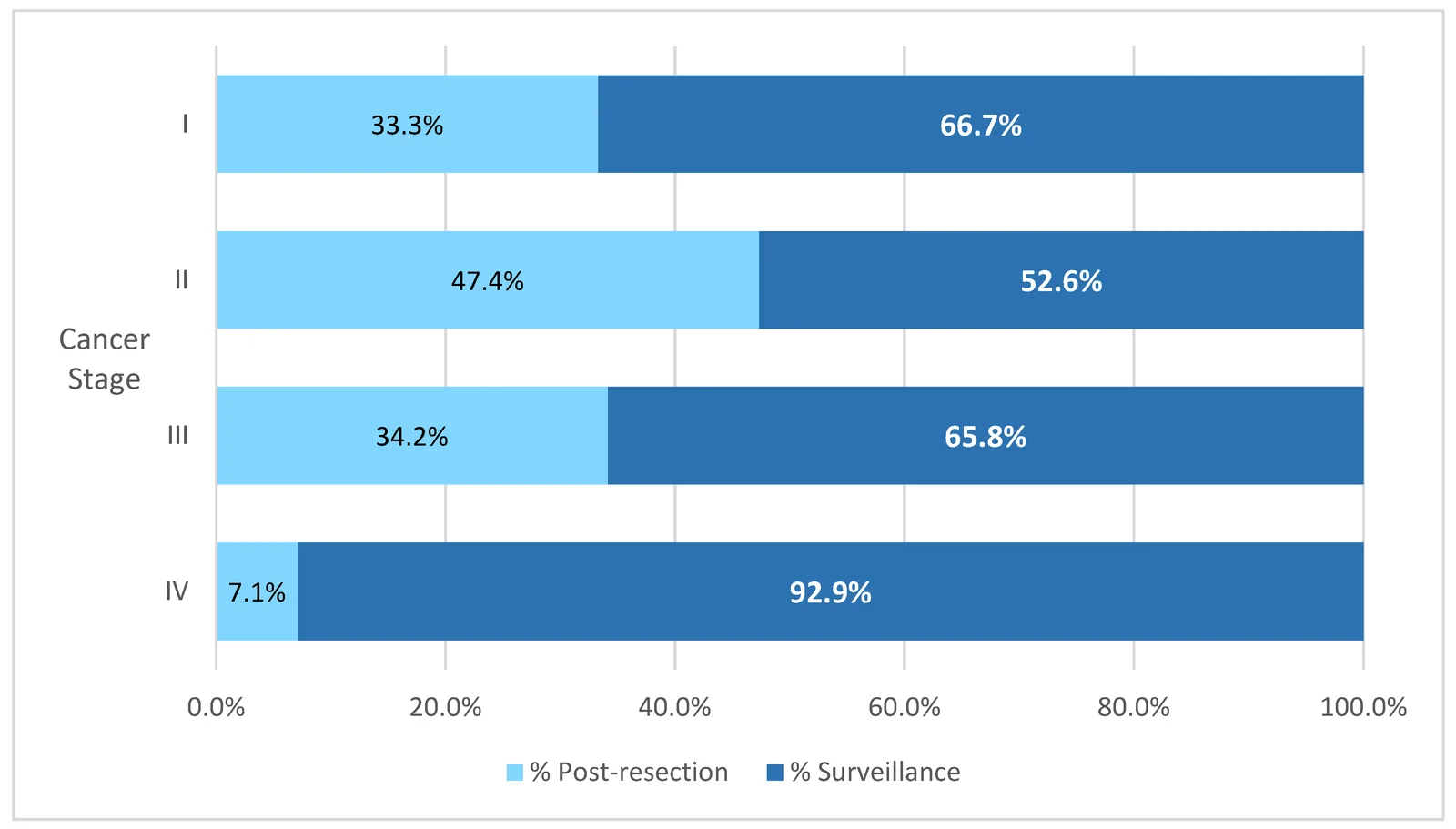
Timing of the first MRD test order for stage I (*n* = 9), stage II (*n* = 57), stage III (*n* = 79), and oligometastatic stage IV (*n* = 14).

For the first MRD test, 66.7% of the tests for stage I patients were performed during surveillance. For patients with stage II disease, 47.4% of first MRD tests were ordered within 12 weeks after surgery, in the post-resection period. More than half of the first MRD tests for stage III patients were ordered during surveillance (>12 weeks after surgery; 65.8%). For patients with oligometastatic stage IV cancer, 92.9% of the first MRD tests were ordered during the surveillance phase (Figure 3).

### 3.5. Median Timing of the MRD Test Order by Cancer Stage

Across all cancer stages, the median time for ordering the first MRD test post-resection was 6 weeks (range 3–12), while the median time for ordering the first surveillance MRD test was 104 weeks (range 13–534) after surgery.

The median time for ordering the first surveillance MRD test for stage I patients was 146.5 weeks (range 55–419). Patients with stage II disease had a median time of 62 weeks (range 13–356) and stage III patients had a median of 118.5 weeks (range 16–337). For patients with oligometastatic stage IV disease, the median time to the first surveillance MRD test was 119 weeks (range 13–534) (Table 2). Overall, the median turnaround time (TAT) from sample receipt to report release was 5 days (range 3–27 days).

## 4. Detailed Case Description

### 4.1. Case 1

**Figure 4:**
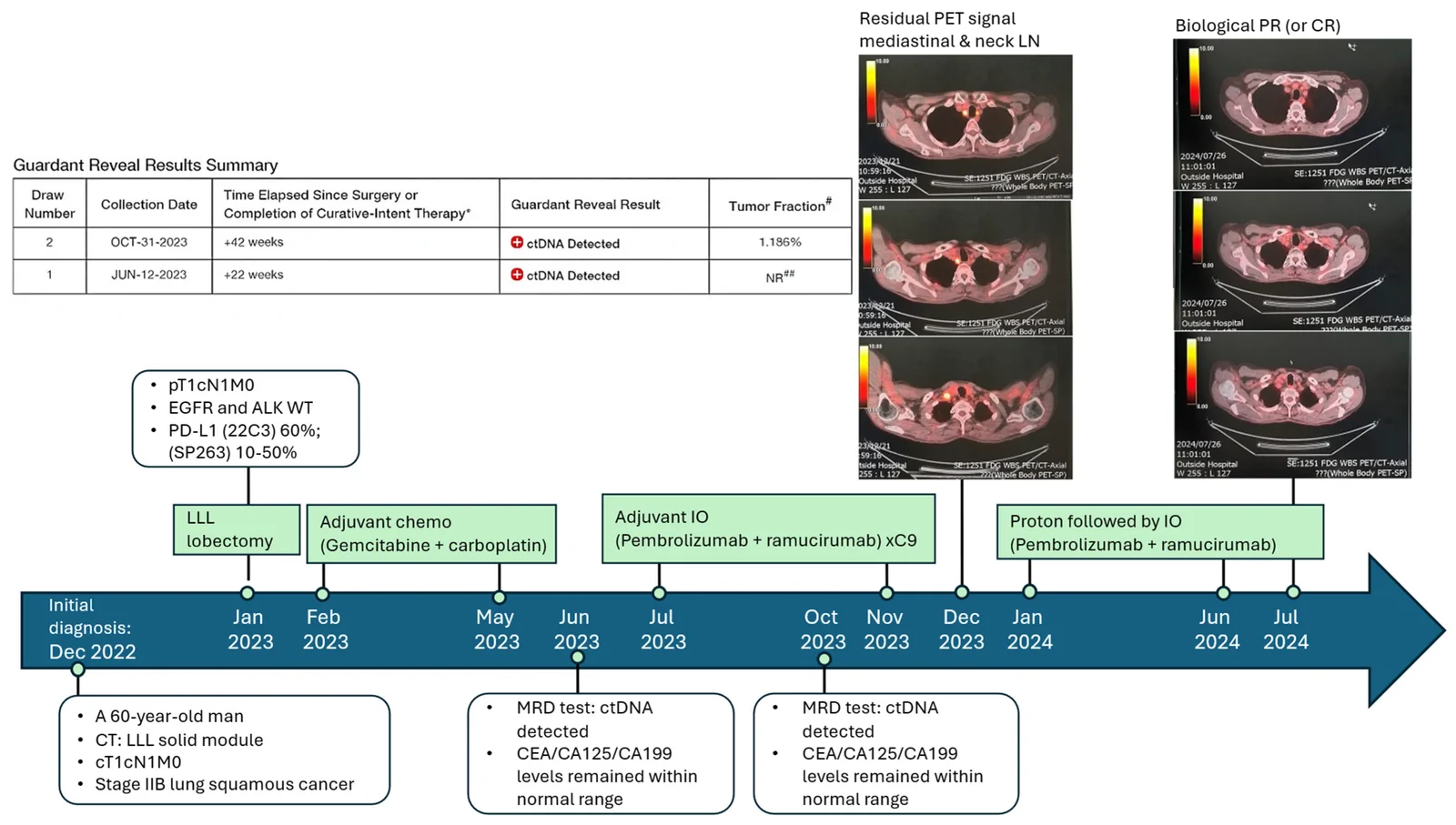
Case 1 timeline of treatment with tissue-free ctDNA MRD results (Guardant Reveal), including the MRD results summary and representative PET/CT images before and after subsequent therapy. ^#^ Tumor fraction is defined as the proportion of tumor molecules present in the cfDNA within the submitted specimen and is based on epigenomic signals. ^##^ Tumor fraction is a feature available on all Reveal tests processed after July 2023. Reveal tests processed before this date will have NR (Not Reported) listed in the Tumor Fraction column.

A Taiwanese male (born 1963) was diagnosed with stage IIB squamous NSCLC and was negative for EGFR and anaplastic lymphoma kinase (ALK) alterations. He underwent curative-intent surgery on 9 January 2023. Tissue-free MRD testing was incorporated into postoperative surveillance using a plasma-only ctDNA assay (Guardant Reveal). At 22 weeks post-surgery, an MRD-positive result was reported. Five days after the MRD-positive result, follow-up imaging confirmed lymph node recurrence, after which additional immunotherapy was administered. At 42 weeks post-surgery, MRD remained positive; proton therapy followed by immunotherapy was subsequently administered. On 26 July 2024, a positron emission tomography (PET) scan showed a partial metabolic response (residual PET signal in mediastinal and neck lymph nodes). MRD test results were considered in treatment modification discussions. The key imaging and MRD timeline is shown in Figure 4.

### 4.2. Case 2

**Figure 5:**
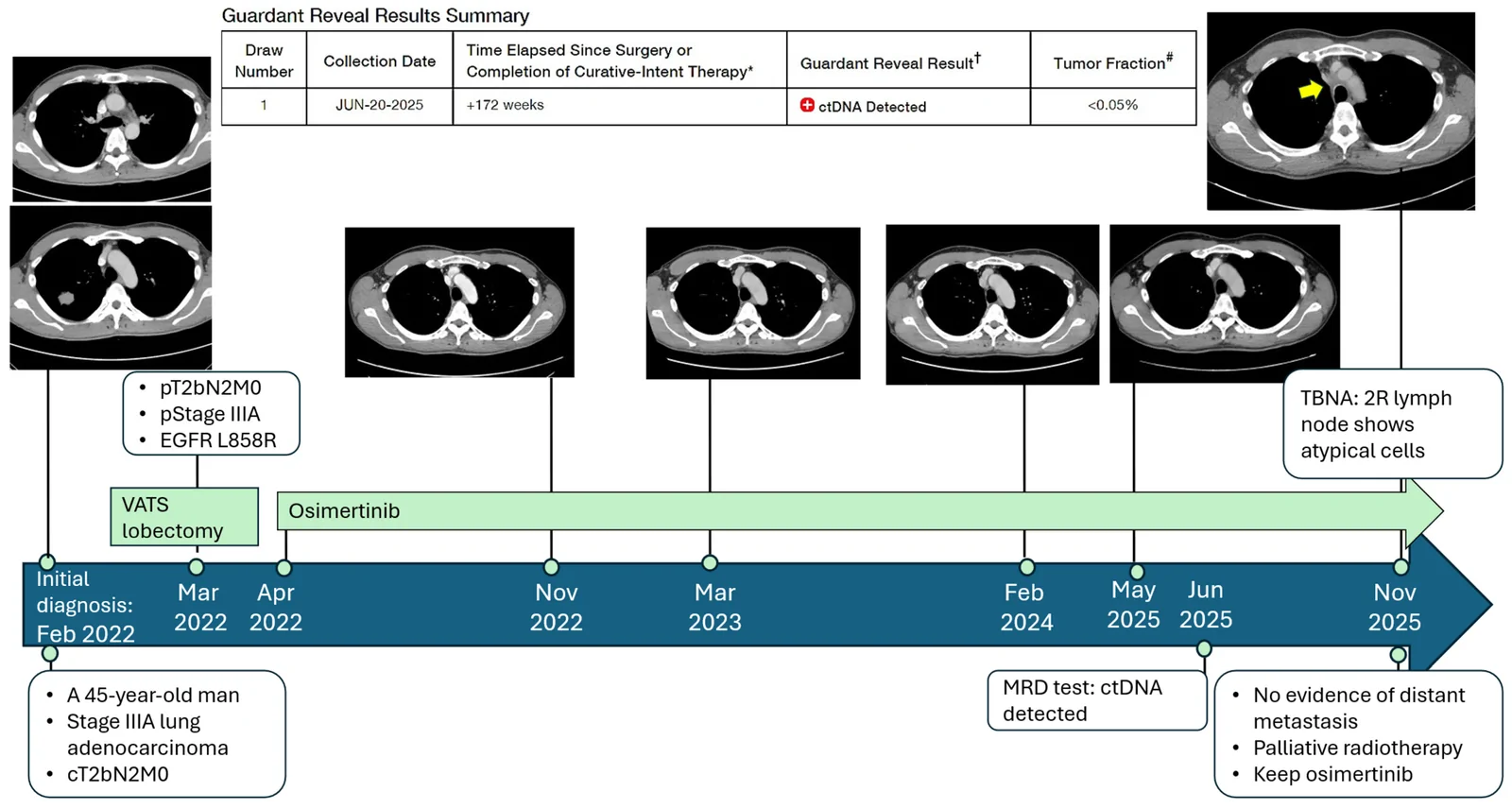
Case 2 timeline of treatment with tissue-free ctDNA MRD results (Guardant Reveal), with representative CT imaging across timepoints and key clinical interventions. VATS, video-assisted thoracoscopic surgery; TBNA, transbronchial needle aspiration. ^†^ Guardant Reveal Result is based on analysis of >2000 differentially methylated regions (DMR) in individuals with cancer compared to individuals without cancer. ^#^ Tumor fraction is defined as the proportion of tumor molecules present in the cfDNA within the submitted specimen and is based on epigenomic signals.

A 46-year-old never-smoking male presented with right upper lobe (RUL) lung adenocarcinoma, clinical stage IIIA (cT2bN2M0, American Joint Committee on Cancer 8th edition). He underwent video-assisted thoracoscopic surgery (VATS) RUL lobectomy, and final pathology confirmed pT2bN2M0, pathological stage IIIA disease. Next-generation sequencing (NGS) identified an EGFR exon 21 p.L858R mutation. After completing platinum-based adjuvant chemotherapy, adjuvant osimertinib therapy was initiated at 80 mg daily at an outside institution. After transfer of care to our institution, he continued osimertinib 80 mg daily and remained without evidence of disease. Treatment was well tolerated, with only grade 1 dry skin and grade 1 folliculitis reported as adverse events. Surveillance imaging, including brain MRI and bone scan, showed no evidence of distant metastasis. At 36 months post-surgery, chest computed tomography (CT) demonstrated no evidence of disease recurrence. After extensive discussion regarding the optimal duration of adjuvant osimertinib therapy, the patient consented to undergo a plasma-only ctDNA assay (Guardant Reveal) to assess molecular residual disease and inform treatment discussions beyond the planned 3-year course. The MRD assay detected ctDNA. This result was considered alongside other clinical information when continuation of osimertinib was recommended. At 42 months post-surgery, routine surveillance chest CT revealed mildly enlarged mediastinal lymph nodes that were concerning for recurrence. Given the clinical suspicion for locoregional relapse, endobronchial ultrasound-guided transbronchial needle aspiration (EBUS-TBNA) was performed targeting the 2R lymph node station. Bronchial washing cytology from the 2R station was positive for malignancy. Definitive pathological examination revealed blood clots containing approximately 15 atypical cells with pleomorphic nuclei. Immunohistochemistry demonstrated positivity for cytokeratin and thyroid transcription factor 1 (TTF-1), confirming recurrent adenocarcinoma. Comprehensive restaging showed no evidence of metastatic disease at other sites. Given the isolated locoregional recurrence, a radiation oncology consultation was obtained, and the patient received radiotherapy to the mediastinal lymph nodes while continuing osimertinib. At 45 months post-surgery, follow-up chest CT demonstrated post-radiation changes in the previously involved mediastinal lymph nodes, with radiographic response to combined-modality therapy. Key imaging and treatment timeline are shown in Figure 5.

### 4.3. Case 3

A 63-year-old male was diagnosed with stage III NSCLC involving bulky and hypermetabolic matted right peribronchial and subcarinal lymph nodes identified on PET-CT. Histopathology from biopsy showed carcinoma; however, immunohistochemical staining was negative for TTF-1 and squamous markers. Based on clinical and radiologic features, the patient was presumed to have lung adenocarcinoma. The disease was considered unresectable after thoracic surgical evaluation.

He received concurrent chemoradiotherapy from May to July 2023, followed by 1 year of durvalumab immunotherapy from September 2023 to September 2024. In November 2024, the patient remained asymptomatic, and serum carcinoembryonic antigen (CEA) levels were within normal limits. However, an MRD test detected ctDNA, with a tumor fraction of 0.204%.

In December 2024, a follow-up PET-CT scan showed no definitive evidence of disease progression, with a subcarinal lymph node measuring 2.04 × 2.91 × 1.79 cm and a standardized uptake value (SUV) of 2.2. Given the low SUV and absence of other active lesions, the findings were interpreted as equivocal.

**Figure 6:**
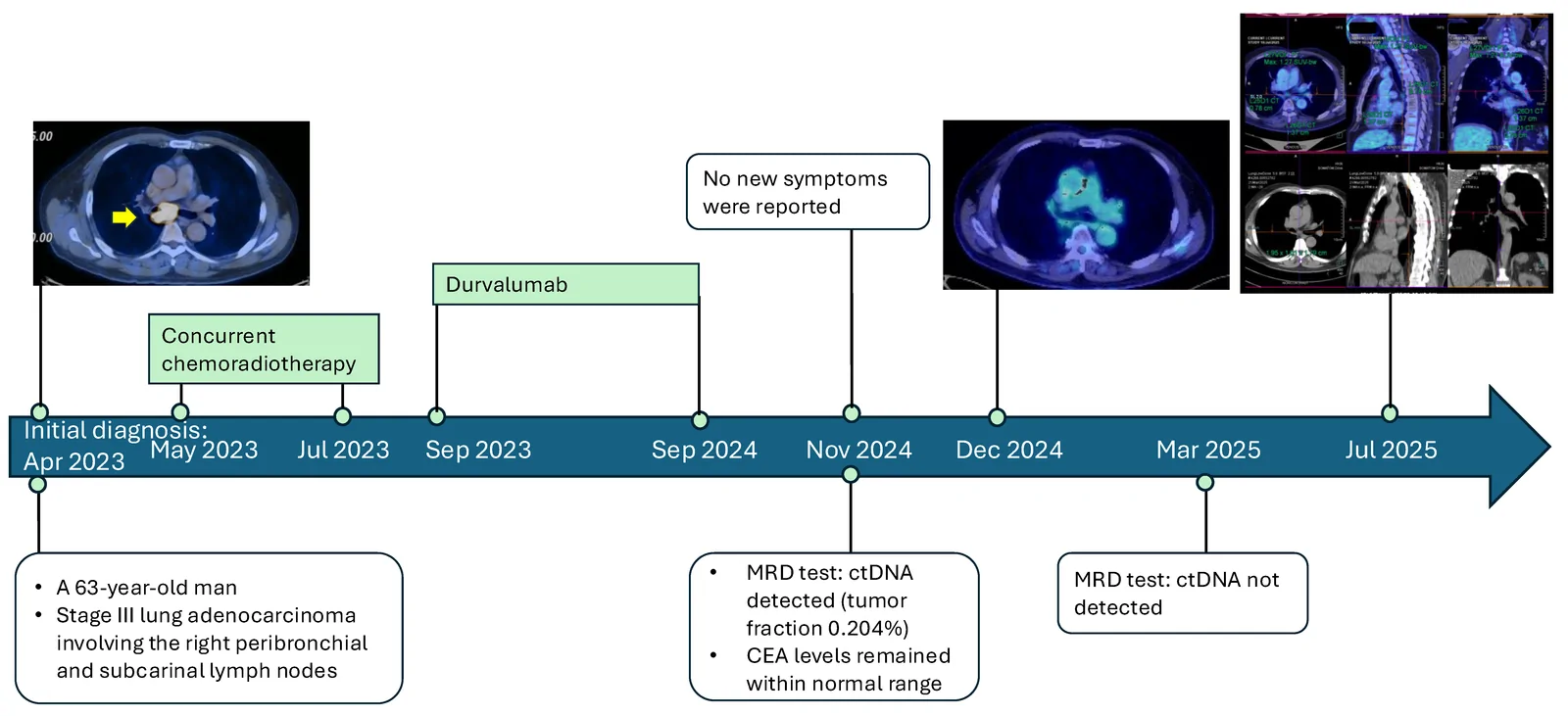
Case 3 timeline of treatment with MRD results (Guardant Reveal). CEA, carcinoembryonic antigen; ctDNA, circulating tumor DNA; MRD, molecular residual disease.

A repeat MRD test in March 2025 showed no detectable ctDNA, indicating conversion to an MRD-negative result. Subsequently, PET-CT imaging in July 2025 demonstrated a reduction in the size and metabolic activity of the subcarinal lymph node (now measuring 1.37 × 0.78 cm, SUV 1.27). The imaging findings aligned with the earlier negative MRD result and were interpreted as consistent with disease control (Figure 6).

This case illustrates the combined use of PET-CT with serial ctDNA testing during post-treatment surveillance in NSCLC. Despite a transient positive MRD result and imaging findings suggestive of possible lymph node activity, the subsequent conversion to negative ctDNA, along with regression of lymphadenopathy, was temporally associated with prior durvalumab therapy but does not establish immune-mediated tumor clearance.

### 4.4. Case 4

A 63-year-old male was diagnosed with stage II (T2N0) lymphoepithelioma-like NSCLC. He underwent video-assisted thoracoscopic right-middle lobectomy in March 2023. Postoperatively, he received four cycles of adjuvant chemotherapy with gemcitabine and carboplatin, completed by July 2023. He remained without evidence of disease through 2024.

In January 2025, surveillance low-dose thoracic CT revealed a few small nodules in the left lung, the largest measuring 0.63 cm. These nodules were stable compared to prior imaging from 2024. The findings were radiologically indeterminate, and Epstein–Barr virus (EBV) DNA testing was negative.

**Figure 7:**
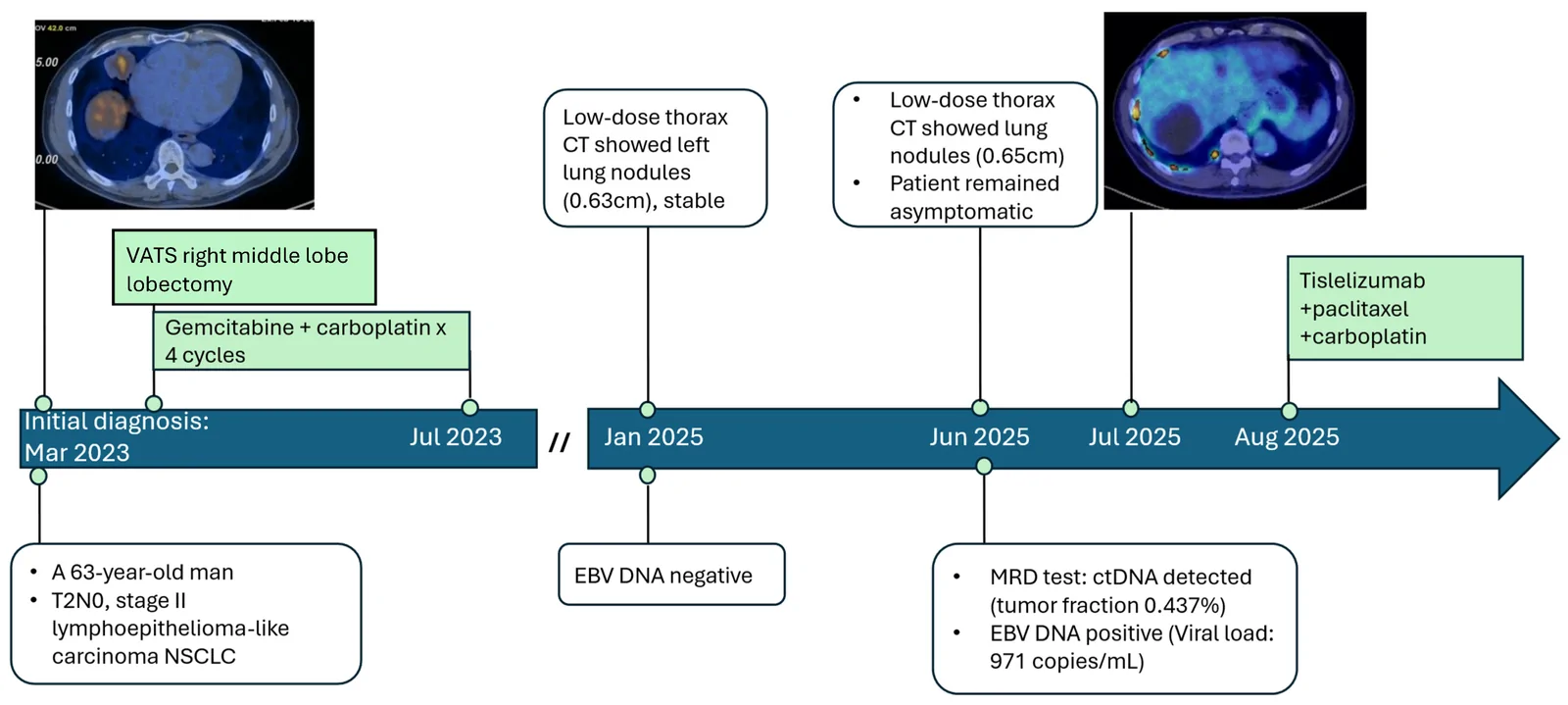
Case 4 timeline of treatment with MRD results (Guardant Reveal). CT, computed tomography; ctDNA, circulating tumor DNA; EBV, Epstein–Barr virus; MRD, molecular residual disease; NSCLC, non-small-cell lung cancer; VATS, video-assisted thoracoscopic surgery.

A repeat low-dose CT in June 2025 again showed stable nodules, now up to 0.65 cm, with a few becoming more conspicuous near the inferior aspect of the right major fissure. Despite the imaging findings, the patient remained asymptomatic. However, EBV DNA testing returned positive with a viral load of 971 copies/mL, considered an equivocal result. Given the equivocal EBV DNA result and stable imaging findings, an MRD test was performed in June 2025. ctDNA was detected with a tumor fraction of 0.437%, raising suspicion for molecular recurrence. This prompted a PET-CT scan in July 2025, which revealed widespread metastatic disease. The patient subsequently started systemic therapy with immunotherapy (tislelizumab) and chemotherapy (paclitaxel and carboplatin) on 20 August 2025 (Figure 7).

In this case, an MRD-positive result prompted PET-CT after indeterminate thoracic imaging; PET-CT subsequently demonstrated metastatic disease. The case illustrates the use of ctDNA testing within a multimodal surveillance strategy in rare cancers such as lymphoepithelioma-like NSCLC.

### 4.5. Case 5

A 52-year-old female was diagnosed with stage IIB NSCLC (pT2aN1) in January 2024. Tissue next-generation sequencing (NGS) identified EGFR L858R and EGFR E709V alterations. She underwent video-assisted thoracoscopic surgery (VATS) right upper lobectomy on 2 February 2024, followed by adjuvant chemotherapy from February to May 2024. The first tissue-free MRD test using a plasma-only ctDNA assay (Guardant Reveal) was performed on 31 May 2024 and showed no detectable ctDNA. This first MRD test served as a molecular baseline for comparison with subsequent tests and was not used for immediate treatment modification.

Adjuvant osimertinib was subsequently initiated in June 2024 and continued through May 2026. Throughout follow-up, serum tumor markers, including CEA and carbohydrate antigen 19-9 (CA19-9), remained within normal limits. These markers therefore provided limited information for surveillance in this case. Serial ctDNA MRD monitoring was incorporated into routine surveillance, with a total of eight MRD tests performed between May 2024 and June 2026.

Surveillance imaging also showed no evidence of recurrence. The most recent brain MRI, performed on 7 March 2026, and chest and abdominal CT, performed on 16 March 2026, demonstrated no evidence of disease (NED).

**Figure 8:**
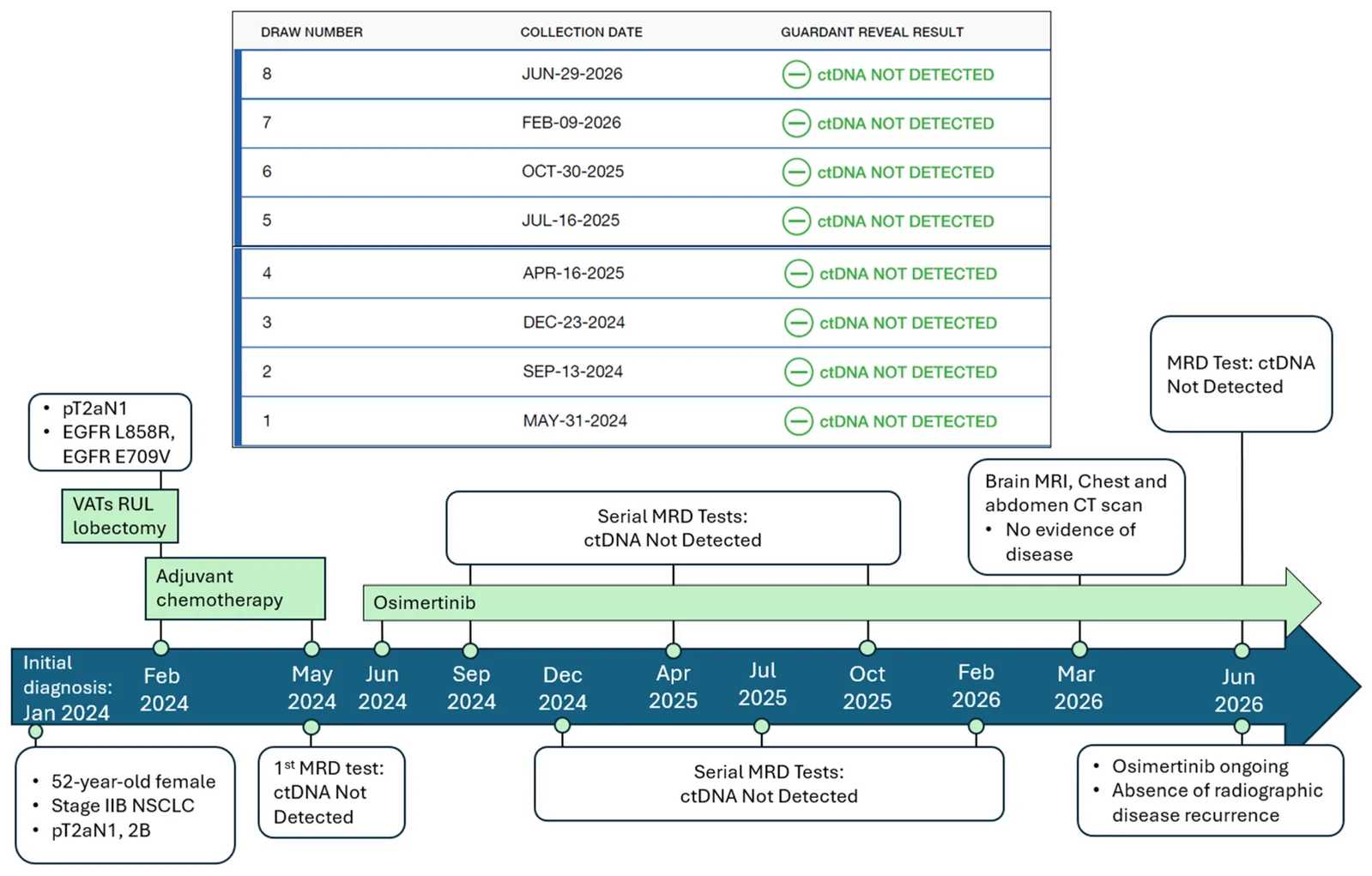
Case 5 timeline of treatment with serial tissue-free ctDNA MRD results (Guardant Reveal). CT, computed tomography; ctDNA, circulating tumor DNA; MRD, molecular residual disease.

This case illustrates the use of serial tissue-free MRD testing alongside routine imaging and biomarker surveillance in a patient with resected EGFR-mutated stage IIB NSCLC receiving adjuvant osimertinib. Persistent ctDNA negativity during longitudinal monitoring was concordant with the continued absence of radiographic recurrence (Figure 8).

### 4.6. Case 6

A 78-year-old male was diagnosed with stage IVB lung adenocarcinoma in February 2024. Baseline molecular profiling did not detect EGFR, ALK, or ROS1 alterations, with a programmed death-ligand 1 (PD-L1) tumor proportion score (TPS) of 60–70%. He received systemic treatment with pembrolizumab for 32 cycles and pemetrexed for 16 cycles, completing this treatment course in February 2026. Following completion of treatment, follow-up PET demonstrated stable disease.

As part of post-treatment assessment, a tissue-free MRD test using a plasma-only ctDNA assay (Guardant Reveal) was performed in March 2026. The assay detected ctDNA with a methylation fraction of 0.197%, despite the absence of radiographic disease progression. Following the stable imaging finding and MRD-positive result, blood-based comprehensive genomic profiling using liquid biopsy NGS (Guardant360) was subsequently performed. The analysis identified a KRAS G12C mutation with a variant allele fraction (VAF) of 0.07%.

**Figure 9:**
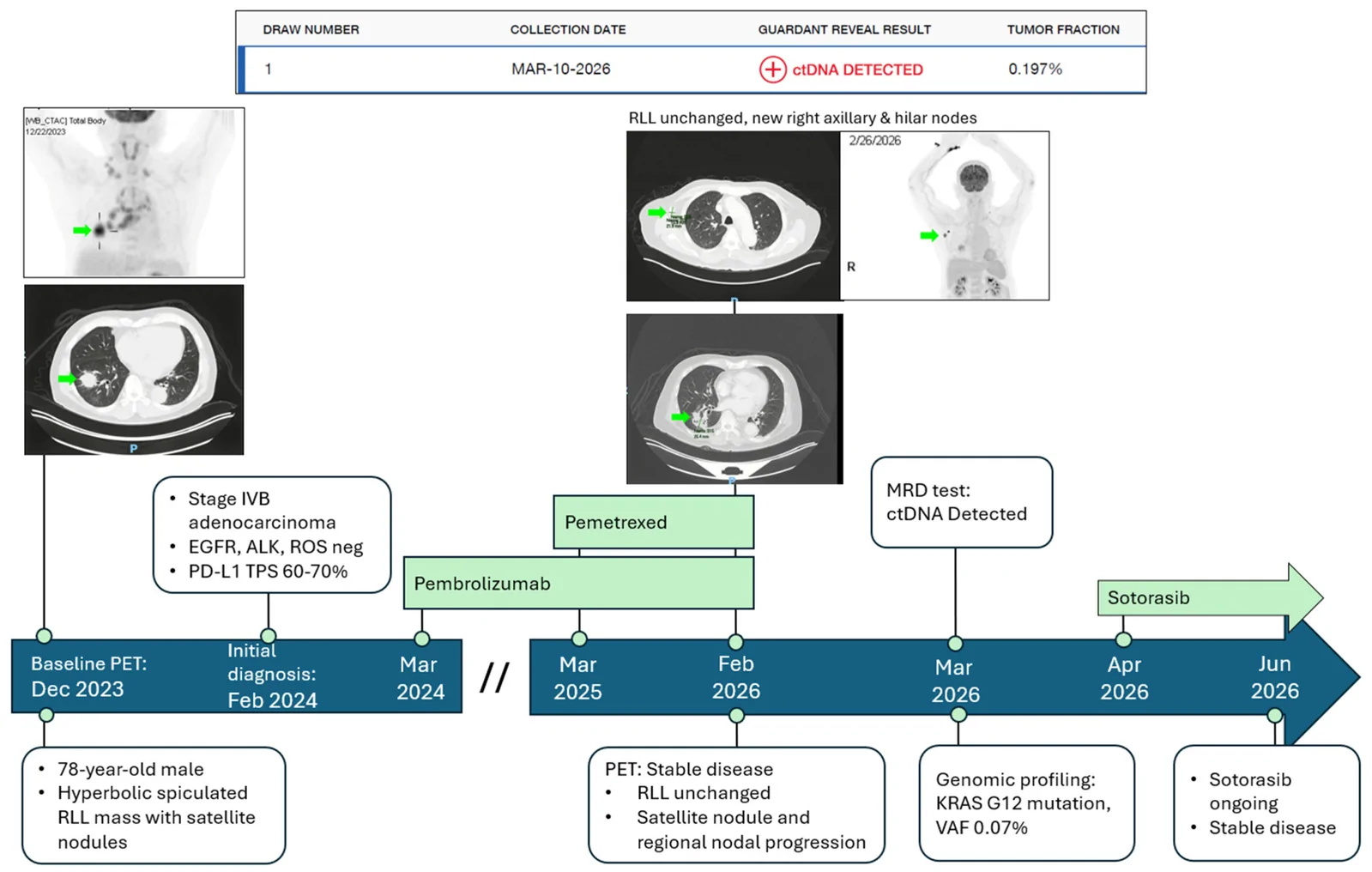
Case 6 timeline of treatment with tissue-free ctDNA MRD results (Guardant Reveal) and subsequent blood-based comprehensive genomic profiling. ctDNA, circulating tumor DNA; MRD, molecular residual disease; PET, positron emission tomography; VAF, variant allele fraction.

After the KRAS G12C alteration was identified, systemic therapy was changed to sotorasib in April 2026. At the most recent follow-up, the patient remained on sotorasib, tolerated treatment well, and had stable disease (Figure 9).

## 5. Discussion

This report combines early real-world utilization data and selected cases to illustrate how tissue-free ctDNA MRD testing is being integrated into NSCLC care in AMEA. Across datasets, clinicians predominantly ordered MRD testing during the surveillance phase rather than only in the immediate postoperative window. Earlier published real-world publication (overlapping snapshot of current cohort) reported that nearly half of NSCLC patients underwent serial testing with a median interval of approximately five months, suggesting that MRD is commonly used as a longitudinal monitoring tool in routine practice. First-test MRD positivity was numerically higher in stages III–IV than in stages I–II; no inferential testing was performed, and differences in testing context may have contributed. The median turnaround time was 5 days; this analysis did not assess its effect on clinical decision-making.

Case 1 of stage IIB squamous NSCLC illustrates a clinical sequence: an MRD-positive result was followed five days later by imaging confirmation of nodal recurrence. Although the lead time over imaging in this case was short (days), molecular relapse can precede radiologic recurrence by months in other settings, and tissue-free MRD may provide an earlier trigger for diagnostic workup, referral to clinical trials, or closer surveillance in selected patients. Case 2 illustrates that an MRD-positive result may occur despite completion of standard adjuvant therapy and should be interpreted in clinical context. However, MRD-guided treatment escalation remains investigational in NSCLC, and prospective studies are needed to define when and how MRD results should influence management.

In Case 3, serial ctDNA results were interpreted alongside standard surveillance measures (imaging and CEA). The sequence included a transient MRD-positive result, equivocal imaging, a subsequent MRD-negative result, and later regression of lymphadenopathy. This temporal pattern is consistent with disease control but does not establish immune-mediated tumor clearance. In Case 4, an MRD-positive result prompted PET-CT after indeterminate thoracic imaging; PET-CT demonstrated metastatic disease. Published lead-time estimates vary by assay, disease setting, sampling schedule, and imaging schedule and should not be inferred from this single case.

Case 5 illustrates the use of ctDNA MRD testing in patients when serum biomarkers remained within normal limits. Persistent MRD-negative results were concordant with the absence of radiographic recurrence, while a positive test may warrant investigations to confirm early recurrences. Case 6 highlights the complementary role of tissue-free MRD testing and subsequent comprehensive genomic profiling in the management of advanced NSCLC. Despite stable surveillance imaging following completion of standard-of-care therapy, an MRD-positive result was followed by further molecular investigation that identified a KRAS G12C alteration and a subsequent change in treatment strategy. The KRAS G12C alteration may have been present at baseline because the pretreatment biomarker assessment did not include KRAS.

Throughout this series, treatment changes that followed an MRD result are reported as a description of what occurred in clinical practice and not as evidence that MRD-guided management improves outcome. In every case, the decision was taken by the treating physician, integrating imaging, symptoms, tumor markers, pathology and patient preference; the MRD result was one input among several, and its independent contribution cannot be isolated. No counterfactual exists for any of these patients, follow-up is short and uncontrolled, and the favorable courses described, such as the partial metabolic response in Case 1 or disease control on sotorasib in Case 6, are attributable to the therapy administered rather than to the act of testing. Conversely, Cases 2 and 4 show that a positive result did not prevent subsequent recurrence or metastatic progression. These narratives are hypothesis-generating; they establish that a result can prompt evaluation, not that prompting evaluation confers benefit.

This analysis is limited by its retrospective design and may have incomplete clinical annotation, selection bias in which patients underwent testing, and limited linkage to outcomes. ctDNA detection can be challenging in low-shedding tumors and early-stage disease, so negative MRD results should not replace guideline-recommended imaging surveillance. In addition, MRD positivity should be interpreted with appropriate clinical context and confirmatory diagnostics, particularly when treatment escalation is being considered. Across all cases, treatment decisions were independently made by the treating physicians, and the assay manufacturer does not endorse or recommend treatment modifications [9].

Four constraints deserve explicit statement, since each bounds what may be concluded. First, there is no comparator group of untested patients and no inferential analysis, so these data are silent on whether MRD testing, or management informed by it, alters recurrence-free or overall survival; the study characterizes test utilization, not clinical benefit, and the two should not be conflated. Second, patients entered the cohort because a clinician chose to order a test, which is a strong and non-random selection: testing is more likely where care is accessible, where reimbursement or self-pay capacity exists, and where the treating clinician was already persuaded of the value of molecular surveillance. The cohort is therefore not representative of the general NSCLC population of the region, and the positivity rates reported here should not be read as population prevalence. Third, the cohort combines stages I-III with oligometastatic stage IV disease and combines post-resection with surveillance testing; these settings differ in biology, treatment intent, baseline recurrence risk and tumor burden, and, consequently, in expected ctDNA shedding. We have reported results stage by stage and testing context by testing context rather than pooling them, and we have reported stages I-III separately from oligometastatic stage IV, but residual heterogeneity within each stratum remains, and small stratum sizes limit what can be said about any of them. Fourth, the contributing territories differ widely in health-system structure, reimbursement of both molecular testing and surveillance imaging, and socioeconomic conditions, so these utilization patterns may not generalize even within AMEA, and still less beyond it.

A balanced appraisal of tissue-free MRD testing versus tissue-informed MRD testing is beyond the scope of the current manuscript, as no comparison was performed. False negatives due to low-shedding tumors or assay limitations are possible.

### Future Perspectives

Two developments would change what can legitimately be claimed for MRD testing in NSCLC. The first is completed prospective trials in which the MRD result itself determines the intervention. Progress here has been uneven, and the recent history is instructive: MERMAID-1 (NCT04385368) and MERMAID-2 (NCT04642469), the two phase III trials designed to randomize MRD-positive patients to adjuvant or surveillance-triggered durvalumab, were both terminated early after perioperative immunotherapy became the standard of care, so neither will answer the escalation question they were built to address [12,13,14,15]. Smaller academic trials continue to test MRD-directed escalation and de-escalation, but the field still lacks a positive randomized trial showing that acting on an MRD result improves survival. Until such a trial reports its findings, MRD-guided treatment modification remains investigational, and an MRD result should not, in isolation, be used to start, stop, extend or intensify therapy. Real-world series such as this one can describe how clinicians are behaving in the interim and can identify the practice patterns a trial would need to accommodate, but they cannot be sufficient evidence on their own.

## 6. Conclusions

In this descriptive AMEA cohort, tissue-free MRD testing was used primarily for post-treatment surveillance rather than in the immediate post-resection window, and was frequently repeated for serial monitoring. First-test positivity was 15.7% (95% CI 10.9–22.2) overall, with wide and mutually overlapping confidence intervals across stages. The accompanying cases illustrate clinical contexts in which an MRD result prompted diagnostic evaluation, but they were purposively selected and, like the cohort analysis, carry no information about assay accuracy or about whether MRD-guided management improves outcome. The contribution of this report is therefore a description of how a tissue-free MRD assay is currently used in a region for which such data have not previously been published [9,10]; it is not evidence of clinical utility, and it should not be cited as such. Prospective, MRD-directed trials with standardized timepoints, thresholds, confirmatory strategies and pre-specified clinical actions remain necessary before MRD results can be used to guide treatment in NSCLC [1,2,13,14,15].

## Figures and Tables

**Table 1:** Demographics of patients.

Patient Characteristic	*n* = 159, (%)
Sex, n (%)	
Male	80 (50.3%)
Female	79 (49.7%)
Median age, years (range)	65 years (range, 28–84)
Cancer stage at first MRD test	
I	9 (5.7%)
II	57 (35.8%)
III	79 (49.7%)
IV (oligometastatic)	14 (8.8%)

**Table 2:** Median timing of MRD testing.

Cancer Stage	Median Time for Ordering Post-Resection MRD Test (Weeks)	Median Time for Ordering First Surveillance MRD Test (Weeks)
I	7 (range 5–8)	146.5 (range 55–419)
II	7 (range 3–11)	62 (range 13–356)
III	5 (range 3–12)	118.5 (range 16–337)
IV (oligometastatic)	3 weeks (*n* = 1)	119.0 (range 13–534)

## Data Availability

De-identified aggregate data supporting the real-world utilization summary are included in this manuscript. Additional details may be available from the corresponding author upon reasonable request, subject to institutional and privacy constraints.

## Abbreviations

The following abbreviations are used in this manuscript:

ALK	Anaplastic lymphoma kinase
AMEA	Asia-Pacific and the Middle East
CA19-9	Carbohydrate antigen 19-9
CEA	Carcinoembryonic antigen
CI	Confidence interval
CRT	Chemoradiotherapy
CT	Computed tomography
ctDNA	Circulating tumor DNA
DFS	Disease-free survival
DMR	Differentially methylated regions
EBUS-TBNA	Endobronchial ultrasound-guided transbronchial needle aspiration
EBV	Epstein–Barr virus
EGFR	Epidermal growth factor receptor
HR	Hazard ratio
MRD	Molecular residual disease
MRI	Magnetic resonance imaging
NED	No evidence of disease
NGS	Next-generation sequencing
NSCLC	Non-small cell lung cancer
OS	Overall survival
PD-L1	Programmed death-ligand 1
PET	Positron emission tomography
PFS	Progression-free survival
RFS	Recurrence-free survival
RUL	Right upper lobe
SUV	Standardized uptake value
TAT	Turnaround time
TF	Methylation tumor fraction
TTF-1	Thyroid transcription factor 1
TPS	Tumor proportion score
VAF	Variant allele fraction
VATS	Video-assisted thoracoscopic surgery
